# Are we developing the right intraoperative AI assistance? Surgeons’ perspectives and desired functions

**DOI:** 10.1007/s00464-026-12791-9

**Published:** 2026-04-09

**Authors:** Franco Badaloni, Gino Kuiper, Ronald de Jong, Romy van Jaarsveld, Yiping Li, Mart van der Neut, Alae Asag-Gau, Adam Zeyara, Marcel Breeuwer, Richard van Hillegersberg, Jelle Ruurda

**Affiliations:** 1 Department of Surgery, University Medical Center Utrecht, Heidelberglaan 100, 3584CX Utrecht, The Netherlands; 2 Department of Biomedical Engineering, Eindhoven University of Technology, Eindhoven, The Netherlands

**Keywords:** Artificial intelligence, Robotic surgery, Anatomy recognition, Intraoperative guidance, Survey

## Abstract

**Background:**

As Artificial intelligence (AI) is increasingly integrated into surgical practice, particularly in robotic surgery, the clinical intraoperative implementation remains limited. Continued progress will require not only technical advances but also a clear understanding of which functions surgeons find valuable in practice. This study aimed to assess surgeons’ perceptions, knowledge, attitudes, and current use of AI-driven intraoperative assistance.

**Methods:**

We conducted a structured, web-based survey of 53 surgeons across 5 continents, assessing demographics, attitudes, knowledge, current use, and perceived usefulness of five AI-based intraoperative guidance components, using video footage from robotic upper gastrointestinal surgeries. Participants were stratified by surgical experience level. Ordinal and categorical data were analyzed using non-parametric tests, and paired comparisons, with statistical significance set at *p* < 0.05.

**Results:**

Perceived knowledge of AI tools for surgery was rated as average or lower by 83.0% of respondents, and 79.2% reported never using such tools intraoperatively. Confidence in relying on clinically validated AI tools was reported by 75.5%, and 86.8% agreed that intraoperative AI assistance could positively impact surgical performance. Anatomy recognition and risk detection received the highest usefulness scores (4.57 ± 0.54 and 4.45 ± 0.72, respectively), followed by vision–language model assistance (3.94 ± 0.97), while step recognition (3.36 ± 1.11) and decision-making guidance (3.51 ± 1.15) were rated lowest; overall usefulness differed significantly across the five components (*p* < 0.001).

**Conclusion:**

This study clarifies how surgeons expect intraoperative AI to be implemented. Despite high perceived usefulness across multiple surgical AI functions, especially for anatomical guidance, adoption in routine practice remains limited, highlighting a gap between positive perceptions and clinical implementation.

**Graphical abstract:**

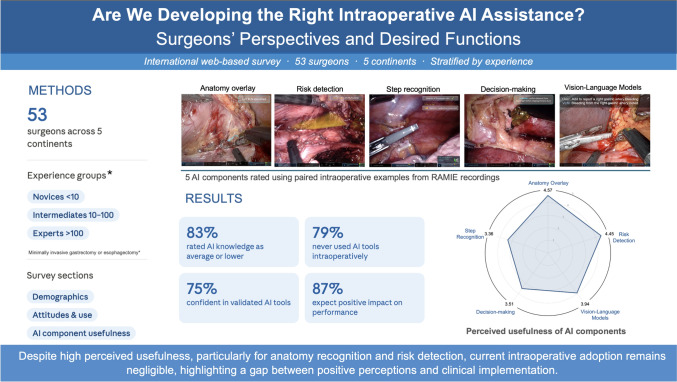

**Supplementary Information:**

The online version contains supplementary material available at 10.1007/s00464-026-12791-9.

Artificial intelligence (AI) is increasingly integrated into surgical workflows, particularly in robotic surgery, with the potential to function as an additional intraoperative tool [1, 2]. Current developmental trajectories indicate an evolution of surgical AI that begins with real-time guidance and may ultimately advance toward the autonomous execution of surgical tasks [1–4].

Right now, surgical AI is capable of real-time interpretation of surgical procedures, with deep learning models that identify anatomical structures, recognize operative steps, detect risks, and support intraoperative decision-making [2, 5–7]. These applications are especially relevant to robot-assisted surgery, where the magnified but confined operative field can challenge spatial orientation and the identification of anatomical structures [8, 9].

However, despite these potential advantages, clinical implementation of real-time intraoperative guidance remains scarce, with most systems confined to experimental or retrospective environments [3]. Continued progress will depend not only on larger video datasets, improved algorithms, and rigorous evaluation frameworks, but also on a clear understanding of the functions that surgeons consider valuable in practice [5, 10].

To help define these requirements, we aimed to assess surgeons’ perceptions, knowledge, attitudes, and current use of AI-driven intraoperative assistance, and to compare these views across different levels of surgical experience.

## Materials and methods

We developed a structured, web-based survey on intraoperative AI tools using footage derived from robot-assisted minimally invasive esophagectomy (RAMIE) recordings. The survey was initially administered during the ESSO Hands-on Course on Minimally Invasive Gastrectomy and Esophagectomy held at the University Medical Center Utrecht in March 2026. Participants were subsequently invited to share the survey with members of their surgical team, enabling broader international distribution. Before starting the questionnaire, participants were provided with a brief explanation of the study’s purpose. Participation was voluntary, and informed consent was obtained from all respondents.

### Survey design

The survey was organized into three sections, with the full questionnaire provided in the Online Appendix.

The first section collected demographic and surgical background information, including age group, country of practice, level of surgical experience, usual surgical approach, and the approximate number of minimally invasive esophagectomy or gastrectomy procedures performed or assisted [10]. The second section evaluated attitudes toward intraoperative AI assistance. Participants rated their knowledge of surgical AI tools, the frequency with which they use such tools intraoperatively, their confidence in relying on clinically validated AI tools (defined as those validated in clinical studies or approved for clinical use), and their expectations regarding the impact of these technologies on surgical practice. Additional questions explored the expected added value of surgical AI, depending on the phase of their career. The third section assessed the perceived usefulness of specific AI components, using footage extracted from RAMIE recordings. For each example, participants were shown paired images: an unaltered intraoperative view and the same surgical view, with an AI-generated overlay. Five applications were evaluated: anatomy recognition, surgical step recognition, risk-structure detection, decision-making assistance, and visual–language model guidance. For each component, participants were asked: “How useful would this component be as part of an integrated intraoperative AI assistance system?” Responses were rated on a 5-point Likert scale ranging from “not useful” to “very useful.”

### Outcome measures

Primary outcome: perceived usefulness of each application of surgical AI (anatomy recognition, surgical step recognition, risk-structure detection, decision-making assistance, and visual–language model guidance).

Secondary outcomes:

- Participants’ knowledge, attitudes, and frequency of use of surgical AI assistance
- Comparison across predefined surgical experience groups

### Statistical analysis

Participants were stratified into three experience groups based on the number of minimally invasive esophagectomy or gastrectomy procedures performed or assisted: experts (> 100 cases), intermediates (10–100 cases), and novices (≤ 10 cases). This categorization is consistent with previous learning-curve thresholds described in previous studies and was used for subgroup analyses [10–12].

Descriptive statistics were used to summarize surgeon demographics and baseline characteristics. Categorical variables were reported as frequencies and percentages. Likert-scale responses [1–5] were treated as ordinal variables and summarized using medians and interquartile ranges (IQR). Differences across groups (surgical experience) were assessed using Kruskal–Wallis or Mann–Whitney U tests for ordinal variables, and chi-square or Fisher’s exact tests for categorical variables. Spearman correlation was used to explore associations between continuous or ordinal variables.

Comparisons across the five applications were assessed using the Friedman test for paired ordinal data. Pairwise differences were explored using Dunn post-hoc testing with Bonferroni correction. Multiple-selection responses were binarized before analysis. For multi-selection items involving repeated binary outcomes within the same respondents, overall differences across categories were assessed using a global paired-proportion approach, with pairwise comparisons adjusted for multiple testing. Participants ranked three reasons for the priority of intraoperative AI (“Improving safety,” “Decision-making support,” and “Increasing confidence”) from 1 (most important) to 3 (least important). Rankings were extracted, summarized as frequencies, and converted into a weighted priority score, where higher scores indicate higher perceived priority.

Statistical significance was defined as *p* < 0.05. All analyses were conducted using Python (pandas, scipy) and R (tidyverse).

## Results

A total of 53 surgeons completed the survey. Most respondents were between 30 and 50 years of age, with 21 (39.6%) aged 30–40 and 15 (28.3%) aged 41–50. The majority were consultant surgeons (60.4%; *n* = 32), followed by trainees/fellows (22.6%; *n* = 12) and residents (17.0%; *n* = 9). Respondents were predominantly from South America (45.3%; *n* = 24) and Europe (43.4%; *n* = 23), collectively representing 19 countries across 5 continents. Case-volume experience in minimally invasive esophagogastric surgery (MIE/MIG) included 32.1% (*n* = 17) of participants with fewer than 10 procedures (novices), 39.6% (*n* = 21) with 10–100 procedures (intermediates), and 28.3% (*n* = 15) with more than 100 cases (experts). Participant demographics and surgical background are summarized in Table 1.

**Table 1:** Participant demographics (*N* = 53)

Variable	*n* (%)
Age	
< 30	8 (15.1%)
30–40	21 (39.6%)
41–50	15 (28.3%)
51–60	6 (11.3%)
> 60	3 (5.7%)
Continent	
South America	24 (45.3%)
Europe	23 (43.4%)
North America	3 (5.7%)
Oceania	2 (3.8%)
Asia	1 (1.9%)
Surgical experience	
Resident	9 (17.0%)
Trainee/fellow	12 (22.6%)
Consultant surgeon	32 (60.4%)
Usual surgical approach	
Thoraco/laparoscopic	36 (67.9%)
Robotic	12 (22.6%)
Open	3 (5.7%)
Hybrid	2 (3.8%)
MIE/MIG case volume	
< 10 cases	17 (32.1%)
10–100 cases	21 (39.6%)
> 100 cases	15 (28.3%)

*MIE/MIG* minimally invasive esophagectomy/gastrectomy

### Knowledge and current use of intraoperative AI tools

**Fig. 1:**
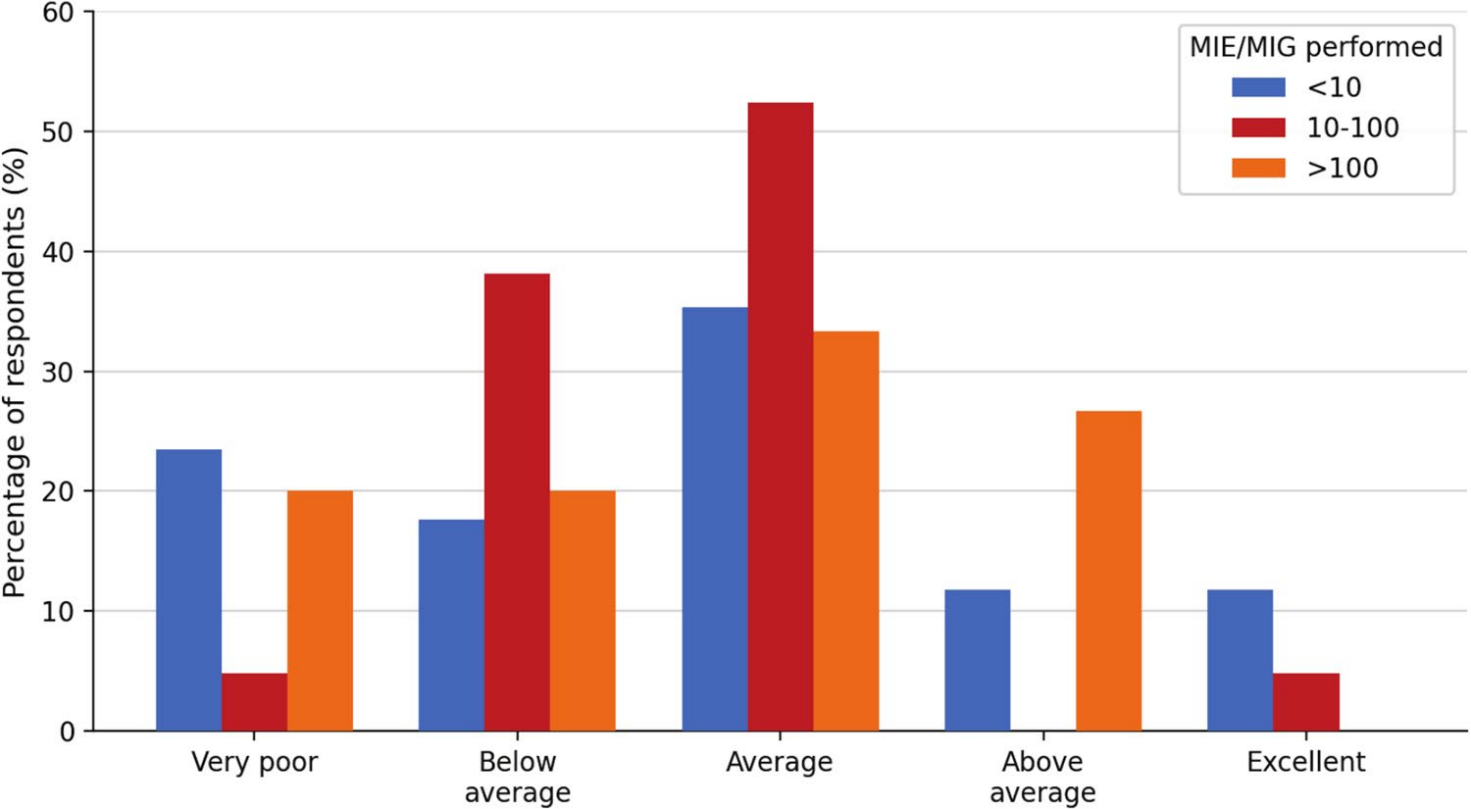
Self-reported knowledge of AI tools for surgery, stratified by surgical experience

Perceived knowledge of AI tools for surgery was rated as average or lower by 83.0% of respondents: 41.5% (*n* = 22) rated their knowledge as *average*, 26.4% (*n* = 14) as *below average*, and 15.1% (*n* = 8) as *very poor*. *Above average* and *excellent* ratings were reported by 11.3% (*n* = 6) and 5.7% (*n* = 3) of the participants, respectively (Fig. 1). Knowledge scores did not differ significantly across experience groups (*p* = 0.938).

**Fig. 2:**
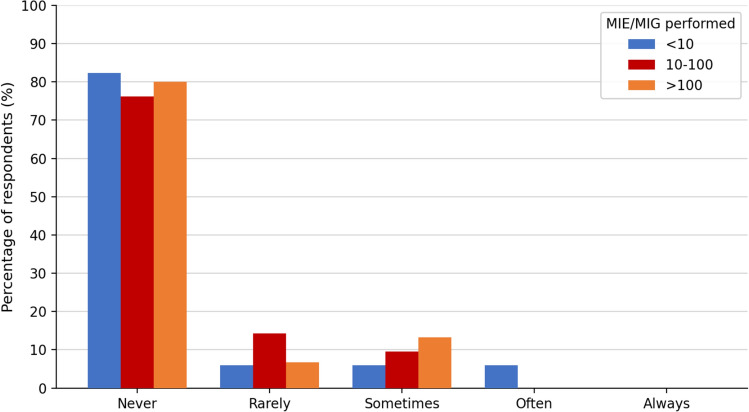
Self-reported frequency of intraoperative surgical AI tool, stratified by surgical experience

Intraoperative use of AI tools was limited across the entire sample: 79.2% (*n* = 42) reported never using such tools during surgery, 9.4% (*n* = 5) rarely, 9.4% (*n* = 5) sometimes, and 1.9% (*n* = 1) often. This pattern was consistent across experience groups, with the proportion reporting no use ranging from 76.2% among intermediates to 82.4% among novices and 80.0% among experts (Fig. 2), and no significant difference observed across groups (*p* = 0.947).

### Confidence in validated intraoperative AI tools

**Fig. 3:**
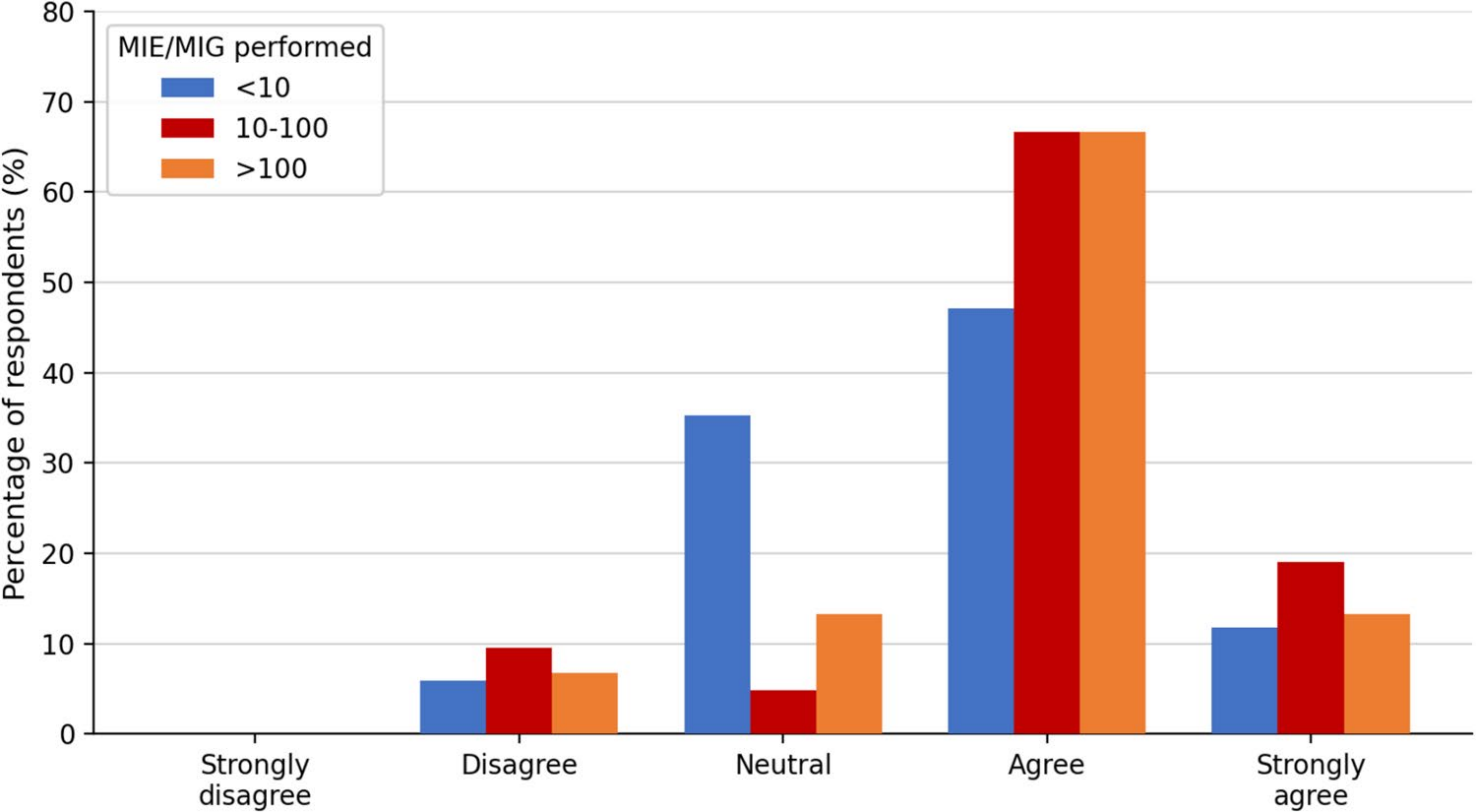
Confidence in relying on clinically validated AI tools during surgery, stratified by surgical experience

Most respondents reported confidence in relying on clinically validated AI tools during surgery: 75.5% (*n* = 40) agreed or strongly agreed, while 17.0% (*n* = 9) were neutral and 7.5% (*n* = 4) disagreed. No respondent strongly disagreed (Fig. 3). Confidence levels were similar across experience groups (*p* = 0.307).

### Perceived impact of intraoperative AI assistance on surgical performance

**Fig. 4:**
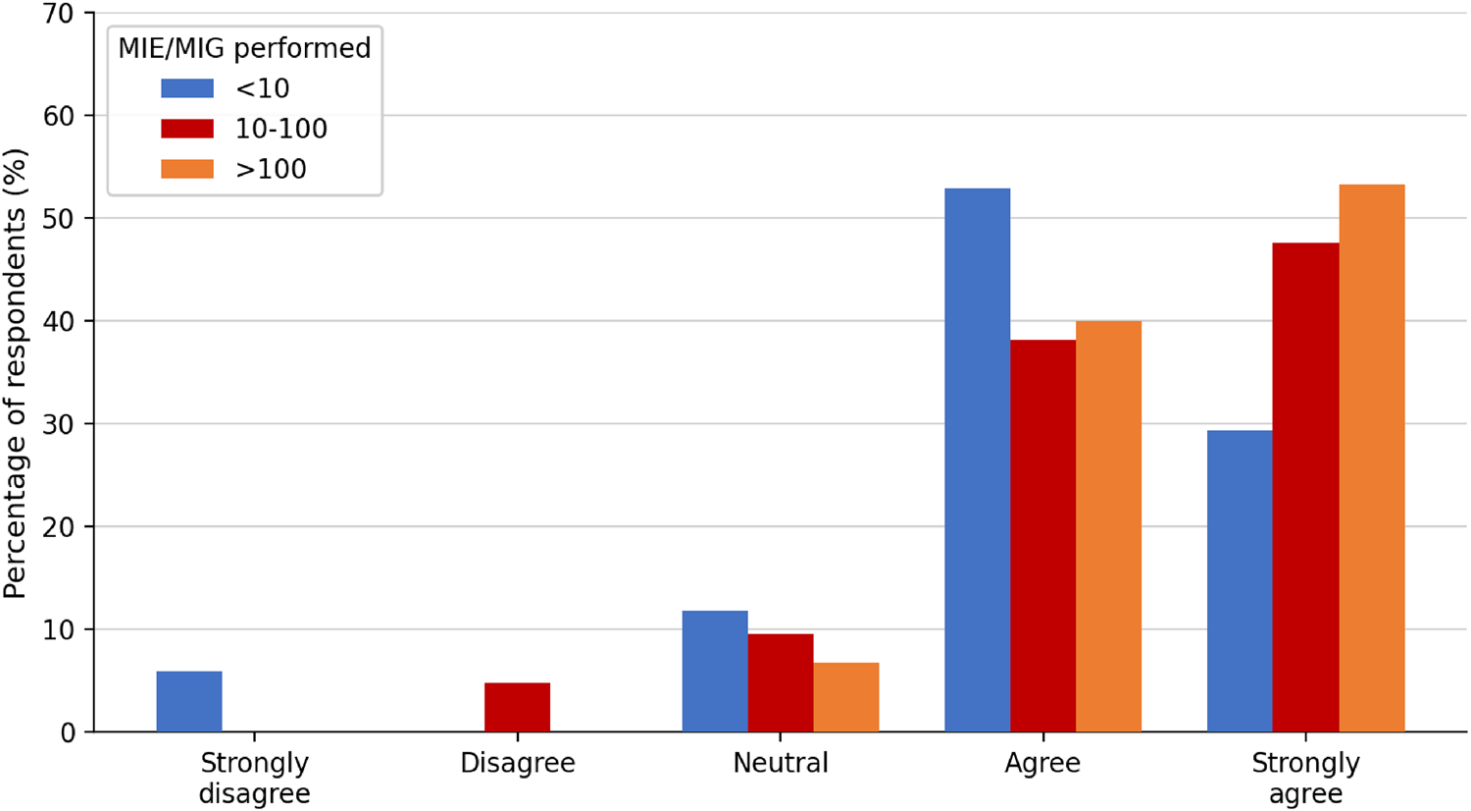
Perceived positive impact of intraoperative AI assistance on surgical performance, stratified by surgical experience

Most respondents agreed or strongly agreed that intraoperative AI assistance could positively impact surgical performance (86.8%; *n* = 46), while 9.4% (*n* = 5) were neutral and 3.8% (*n* = 2) disagreed or strongly disagreed (Fig. 4). Agreement was numerically highest among experts (93.3%; 14/15), followed by intermediates (85.7%; 18/21) and novices (82.4%; 14/17). No significant difference was observed across experience groups (*p* = 0.328).

**Fig. 5:**
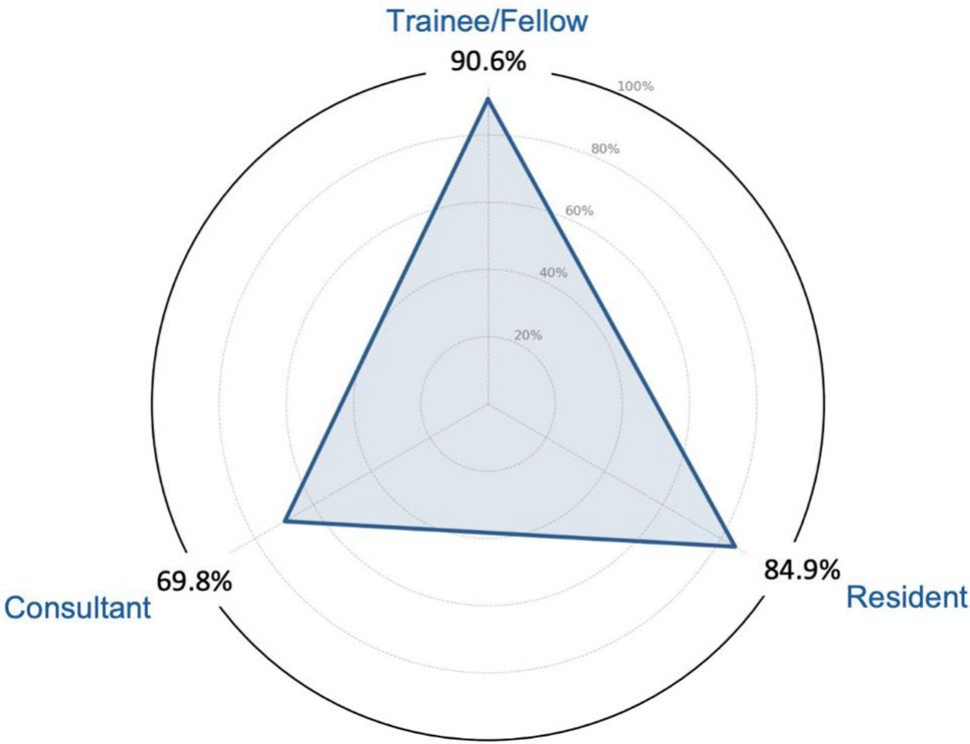
Radar plot depicting perceived stages of surgical training at which intraoperative AI assistance would be most useful. Percentages exceed 100% because multiple selections were allowed

When assessing at which stage of surgical training intraoperative AI would be most beneficial, respondents most frequently selected the trainee/fellow level (90.6%; *n* = 48), followed by residents (84.9%; *n* = 45) and consultant surgeons (69.8%; *n* = 37) (Fig. 5). The proportion selecting the trainee/fellow level was significantly higher than those selecting the consultant level (*p* = 0.029), while no significant difference was found between trainee/fellow and resident, or between resident and consultant.

**Fig. 6:**
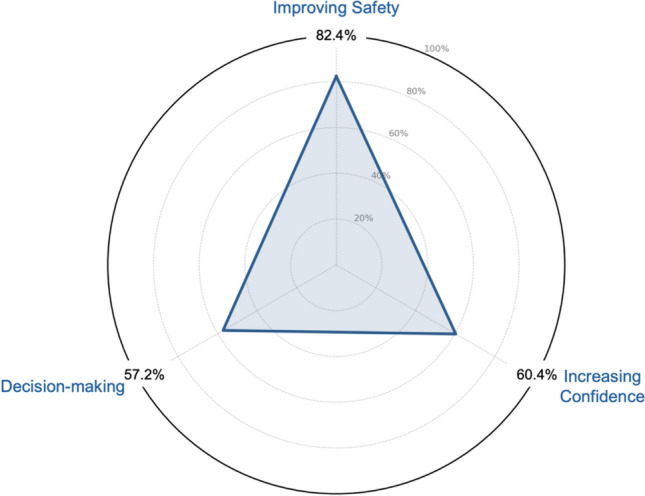
Radar plot of weighted priority scores for the perceived benefits of intraoperative AI assistance

Improving safety was the most frequently selected first-priority objective (56.6%; *n* = 30), with increasing confidence and decision-making support as secondary priorities, reflected in their weighted scores (82.4%, 60.4%, and 57.2%, respectively) (Fig. 6).

### Usefulness of AI-assisted intraoperative functions

**Fig. 7:**
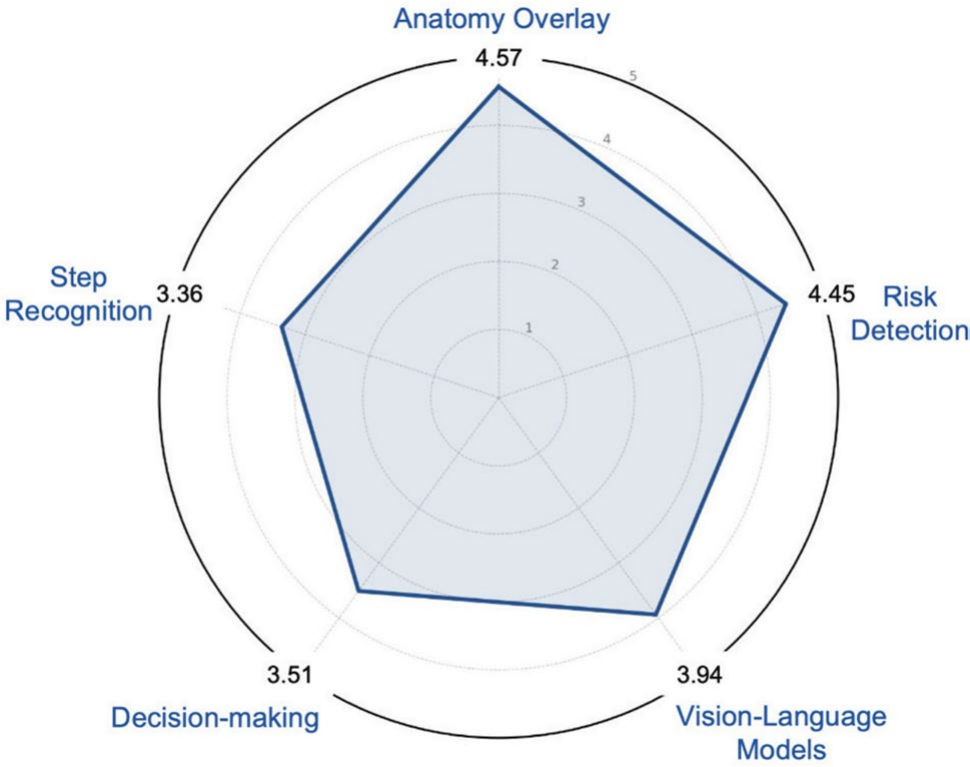
Radar plot of perceived usefulness of intraoperative AI support tools

Participants rated the usefulness of each AI-assisted intraoperative function on a 5-point Likert scale (1 = not useful, 5 = very useful). Anatomy recognition received the highest mean score (4.57 ± 0.54), followed by risk detection (4.45 ± 0.72), vision–language model assistance (3.94 ± 0.97), decision-making guidance (3.51 ± 1.15), and step recognition (3.36 ± 1.11) (Fig. 7). Usefulness ratings differed significantly across the five components (*p* < 0.001). Both anatomy recognition and risk detection were rated significantly higher than vision–language model assistance, decision-making guidance, and step recognition (all *p* < 0.05 after correction). Vision-language model assistance was also rated significantly higher than both decision-making guidance and step recognition. No significant difference was found between anatomy recognition and risk detection, or between decision-making guidance and step recognition.

## Discussion

This study offers one of the first structured assessments of surgeon perspectives on intraoperative AI assistance in robotic surgery, bridging the gap between algorithm design and the support surgeons consider most useful.

Our findings indicate that surgeons are willing to employ a wide range of intraoperative AI tools, although their preferences vary depending on the type of assistance provided. The perceived usefulness of the evaluated functions followed a clear gradient. Anatomy recognition occupied the upper end of this spectrum, followed by risk-detection and vision–language model guidance. Decision-making assistance and step-recognition support were placed toward the lower end of the gradient yet both remained within the range of functions considered useful by most respondents. Overall, these results indicate that surgeons value all intraoperative AI functions assessed, while favoring tools that enhance anatomical recognition over those oriented toward procedural segmentation or decision-making.

Beyond the perceived usefulness of individual functions, our results reveal a gap between surgeons’ attitudes and their current use of AI applications. More than 86% of respondents agreed that intraoperative AI assistance could positively impact surgical performance, particularly during early stages of training, and 75.5% expressed confidence in relying on clinically validated tools. By contrast, 79.2% reported never using intraoperative AI tools during surgery. This mismatch suggests that current applications may still lack the availability, accessibility, integration, and reliability required for routine clinical use. Recent studies have attempted to explain this gap, consistently citing persistent barriers related to workflow integration, training requirements, system costs, and the need for robust oversight and validation in real-world settings [13–15].

From a translational perspective, these findings allow preliminary recommendations regarding how preferred AI functions could be integrated into intraoperative workflows. Overall, surgeons appear to envision intraoperative AI primarily as an assistive, context-aware system rather than a decision-maker. Highly ranked functions such as anatomy recognition and risk detection emerge as the most clinically relevant entry points for implementation, as they directly support safety and anatomical understanding during critical surgical steps. In contrast, lower-ranked functions, including surgical step recognition and decision-making assistance, seem to be perceived as complementary features that may be best implemented as optional or on-demand tools, thereby limiting unnecessary interruptions and cognitive load. The consistently high ratings for vision–language model assistance further indicate interest in flexible user-driven interaction, reinforcing a model of intraoperative AI that prioritizes safety, usability, and surgeon control. Taken together, these findings support the development of a modular, integrated intraoperative AI assistance system in which individual components can be selectively activated based on surgical context, experience level, and clinical need.

### Findings in context

Experience-based stratification showed that surgeons across all experience levels consistently recognized the usefulness of intraoperative AI, in contrast to prior work by de Jong et al., which reported expertise-dependent variation in how AI outputs are interpreted, particularly in anatomy-segmentation tasks [10]. In our study, no significant difference in attitudes, confidence, or perceived usefulness was observed across experience groups. The uniformly low rates of intraoperative AI use across all groups further suggest that limited access and availability, rather than experience-dependent factors, may be the primary barrier to adoption. Although the survey question specifically referred to intraoperative surgical AI tools, the possibility that some respondents interpreted this broadly cannot be entirely excluded.

Most survey studies on AI in surgery evaluate surgeons’ expectations in domains such as diagnosis or perioperative management rather than examining intraoperative implementation in the context of robotic surgery [15–18]. Nevertheless, they consistently report positive attitudes toward AI, a pattern that aligns with the optimism observed in our cohort despite the differing clinical scope. In another survey study, Pecqueux et al. found that most participants rated their own knowledge as average or rudimentary and acknowledged limited use of AI tools in their clinical environment [17]. This combination of favorable attitudes, average self-reported knowledge, and restricted implementation is consistent with our findings.

### Strengths and limitations

Several limitations of this study should be acknowledged. First, although the sample was expanded through a snowball distribution strategy, recruitment was initiated at a single minimally invasive techniques course, which may have introduced selection bias favoring surgeons with prior interest in implementing new technologies. Even so, the cohort encompassed the full spectrum of training levels, ensuring representation from residents to senior experienced consultants, and represented 19 countries across 5 continents, reflecting broad international participation. A further limitation is that respondents with limited knowledge on AI may have held inaccurate or incomplete conceptualizations of intraoperative AI, which could have influenced how they interpreted the presented intraoperative functions, as suggested in previous surveys [16]. Moreover, the questionnaire was developed specifically for this study, and its measurement validity was not formally assessed. Finally, AI functions were assessed using static images, which may not reflect how surgeons interpret such tools during live procedures. Further research should assess these functions in real-time surgical settings to better define their practical clinical value and generalizability.

Irrespective of these limitations, our study helps fill a gap repeatedly highlighted in prior work: the need to understand how surgeons want intraoperative AI to be implemented and which functions they consider most valuable for real-time use [10, 16]. Defining these requirements provides a foundation for future development, guiding the creation of AI tools that can be effectively integrated into the operative field.

In conclusion, surgeons across experience levels highly value intraoperative AI assistance, particularly for anatomy recognition. Current limited adoption likely reflects barriers of availability and implementation rather than lack of interest, and defining these preferences provides a foundation for developing clinically meaningful AI tools.

## Supplementary Information

Below is the link to the electronic supplementary material.

Supplementary file1 (DOCX 17 kb)

Supplementary file2 (DOCX 3138 kb)

Supplementary file3 (DOCX 4824 kb)

Supplementary file4 (DOCX 7142 kb)

Supplementary file5 (DOCX 3895 kb)

Supplementary file6 (DOCX 1338 kb)

## Data Availability

Data associated with this study is available from the corresponding author upon request.
