# Supplementary material for: Are we developing the right intraoperative AI assistance? Surgeons’ perspectives and desired functions
Source: Surg Endosc. 2026 Apr 9;40(6):5259–66. doi: 10.1007/s00464-026-12791-9 (PMC13246846; doi:10.1007/s00464-026-12791-9)
Supplement: Supplementary file 1 — Supplementary file1 (DOCX 17 kb) [file 464_2026_12791_MOESM1_ESM.docx]

**Appendix: Survey Design**

**Title:** Expectations & Involvement with intraoperative AI assistance

*Dear Participant,*
*Thank you for taking part in this survey on the use of artificial intelligence (AI) during surgery. The purpose of this study is to understand surgeons’ perceptions, knowledge, attitudes, and current use of AI-driven intraoperative assistance.* *The survey should take 7 minutes to complete.*

**Section 1: Experience**

**1.1 What is your age?** <30/ 30-40/ 41-50/ 51-60/ 60+

**1.2 In which country are you currently practicing surgery?**

**1.3 Surgical qualification:** Resident / Trainee or Fellow / Consultant surgeon

**1.4 Usual approach:** Open / Thoraco-Laparoscopic / Hybrid/ Robotic

**1.5 How many minimally invasive gastrectomies or esophagectomies have you assisted or performed?** <10/ 10-100/ >100

**Section 2: Involvement, expectations & current use***Please consider that this survey refers specifically to intraoperative AI used during surgery (AI tools designed for surgical use)*

**2.1 How would you rate your knowledge of surgical AI assistance tools?**
 Very poor / Below average / Average / Above average / Excellent

**2.2 How often do you use an intraoperative surgical AI assistance tool during surgery?**
Never / Rarely / Sometimes / Often / Always

**2.3 Do you think intraoperative AI assistance could positively impact performance during surgery? (e.g. anatomical interpretation, intraoperative decision-making, safety, surgeon's confidence)**
Strongly disagree / Disagree / Neutral / Agree / Strongly agree

**2.4 How confident would you be in relying on clinically validated AI tools during surgery? (validated in clinical studies or approved for clinical use)**
0= Not confident/ 5=Very confident

**2.5 At which stage of surgical training do you consider intraoperative AI assistance can be most useful? (You may select more than one option)**
None/ Resident/ Trainee or Fellow/ Consultant surgeon/ All

**2.6 What is the main reason surgical AI assistance would be useful to you intraoperatively?**
*Please arrange the following items in your preferred order.*
1: Improving safety/ 2: Decision-making/ 3: Increasing confidence

**Section 3: Usefulness**
*You will be shown examples of potential components of an integrated intraoperative AI assistance system. Each function is presented separately to assess its perceived usefulness from a user perspective.*

**3.1 Anatomy recognition:** identification and highlighting of anatomical structures in real time.

<Figure 8>
**Example**: Intraoperative image of a RAMIE, showing the left paratracheal dissection. The left recurrent laryngeal nerve is highlighted.

**How useful would this component be as part of an integrated intraoperative AI assistance system?**
Likert scale: 0 = Not useful, 5 = Very useful

**3.2 Risk-detection:** Context-aware detection of structures or zones at risk (e.g., nerves, vascular injury), based on anatomical recognition and spatial proximity.

<Figure 9>
**Example**: Intraoperative image of a RAMIE, showing the left paratracheal dissection. An overlay highlights the left recurrent laryngeal nerve zone, as a warning.

**How useful would this component be as part of an integrated intraoperative AI assistance system?**
 Likert scale: 0 = Not useful, 5 = Very useful

**3.3 Step recognition:** Automatic identification of the current surgical step or phase.

<Figure 10>
***Example****: Intraoperative image of a robotic esophagectomy, immediately after the division of the azygos vein. AI assistance suggests the next step, superior mediastinal dissection.*

**How useful would this component be as part of an integrated intraoperative AI assistance system?**

Likert scale: 0 = Not useful, 5 = Very useful

**3.4 Decision-making assistance**: context-based intraoperative guidance during specific surgical moments. The AI model first recognizes the current surgical phase or step and based on that context, provides targeted reminders or alerts regarding key anatomical or procedural considerations.

<Figure 11>
**Example**: AI-assisted intraoperative image of thoracic duct dissection during RAMIE, emphasizing the need to verify safe clipping level relative to the diaphragm.

**How useful would this component be as part of an integrated intraoperative AI assistance system?**
Likert scale: 0 = Not useful, 5 = Very useful

**3.5 Surgical vision-language models:** Multimodal AI systems combining visual and textual data for intraoperative guidance (like Siri or Alexa, it acts like a voice assistant), enabling question–answering, documentation, contextual guidance* and education.

<Figure 12>

**Examples** of vision–language model assistance during RAMIE: operative report annotation, procedural step timing, and real-time response to user query.

** The same AI functions (e.g., anatomy recognition or phase recognition) could be triggered via a manual interface (on-off bottom), or through a vision–language model.*

**How useful would this component be as part of an integrated intraoperative AI assistance system?**
Likert scale: 0 = Not useful, 5 = Very useful
