# Supplementary material for: Are we developing the right intraoperative AI assistance? Surgeons’ perspectives and desired functions
Source: Surg Endosc. 2026 Apr 9;40(6):5259–66. doi: 10.1007/s00464-026-12791-9 (PMC13246846; doi:10.1007/s00464-026-12791-9)
Supplement: Supplementary file 2 — Supplementary file2 (DOCX 3138 kb) [file 464_2026_12791_MOESM2_ESM.docx]

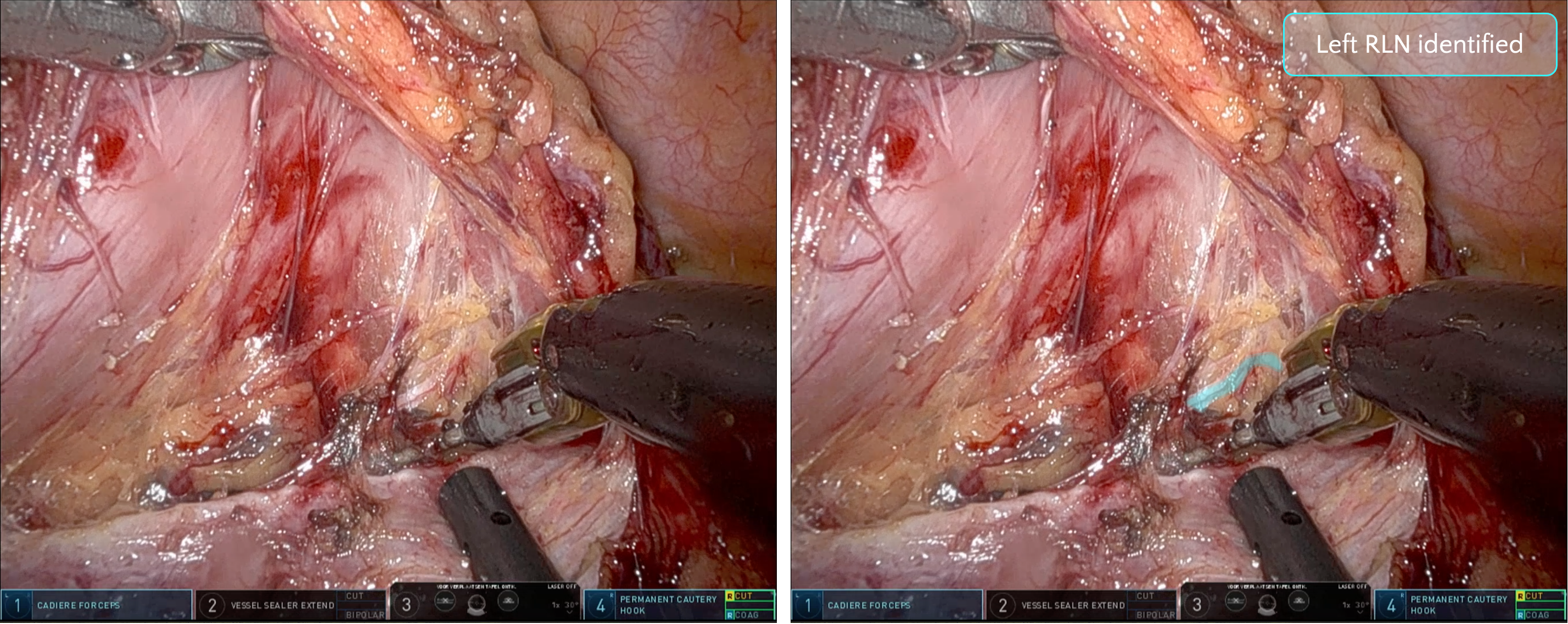

**Figure 8.** Intraoperative image of a RAMIE, showing the left paratracheal dissection. The left recurrent laryngeal nerve is highlighted.
