# Supplementary material for: Are we developing the right intraoperative AI assistance? Surgeons’ perspectives and desired functions
Source: Surg Endosc. 2026 Apr 9;40(6):5259–66. doi: 10.1007/s00464-026-12791-9 (PMC13246846; doi:10.1007/s00464-026-12791-9)
Supplement: Supplementary file 4 — Supplementary file4 (DOCX 7142 kb) [file 464_2026_12791_MOESM4_ESM.docx]

**
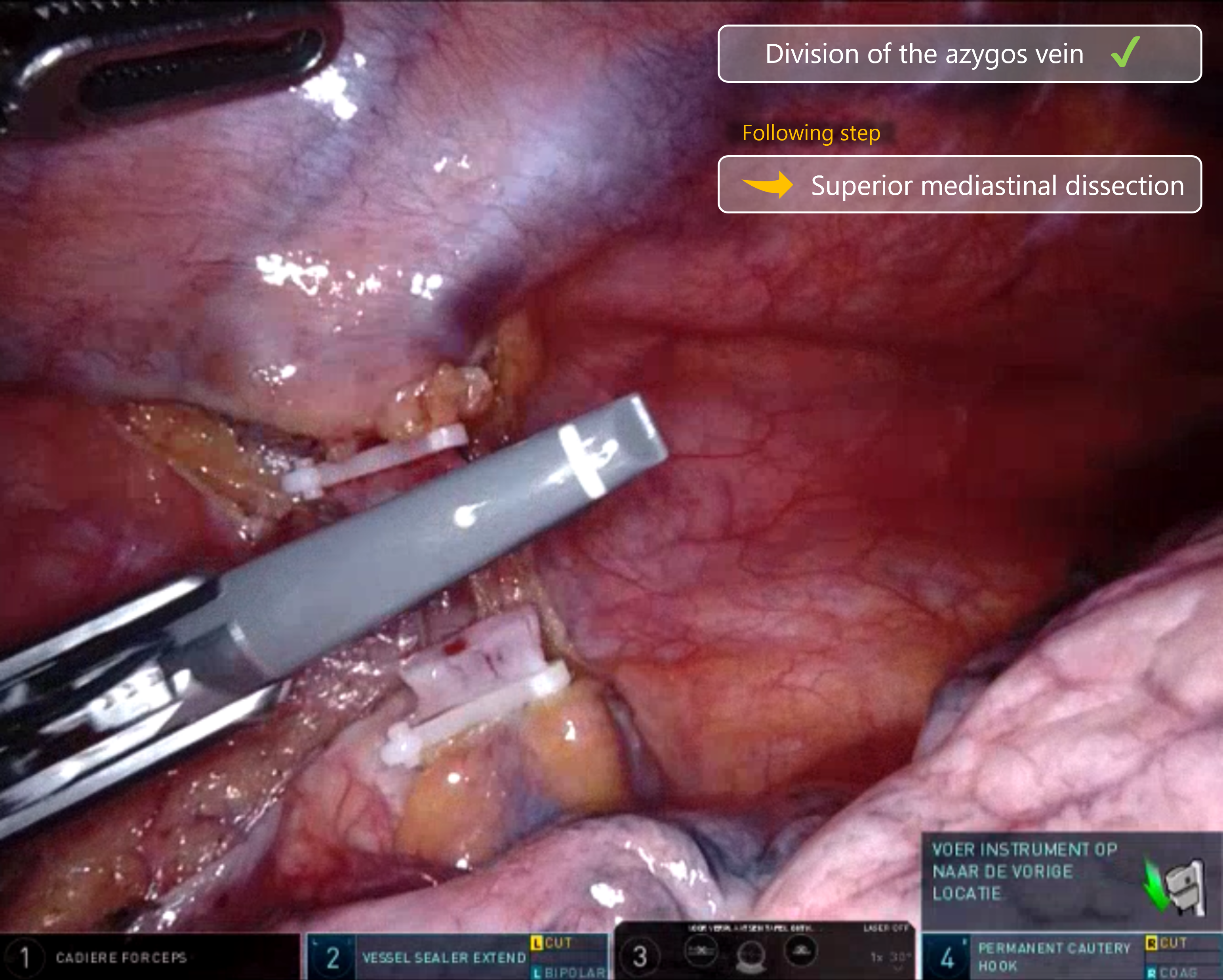

Figure 10.** Intraoperative image of a robotic esophagectomy, inmediately after the division of the azygos vein. AI assistance suggests the next step, superior mediastinal dissection.
