# Supplementary material for: Are we developing the right intraoperative AI assistance? Surgeons’ perspectives and desired functions
Source: Surg Endosc. 2026 Apr 9;40(6):5259–66. doi: 10.1007/s00464-026-12791-9 (PMC13246846; doi:10.1007/s00464-026-12791-9)
Supplement: Supplementary file 5 — Supplementary file5 (DOCX 3895 kb) [file 464_2026_12791_MOESM5_ESM.docx]

**
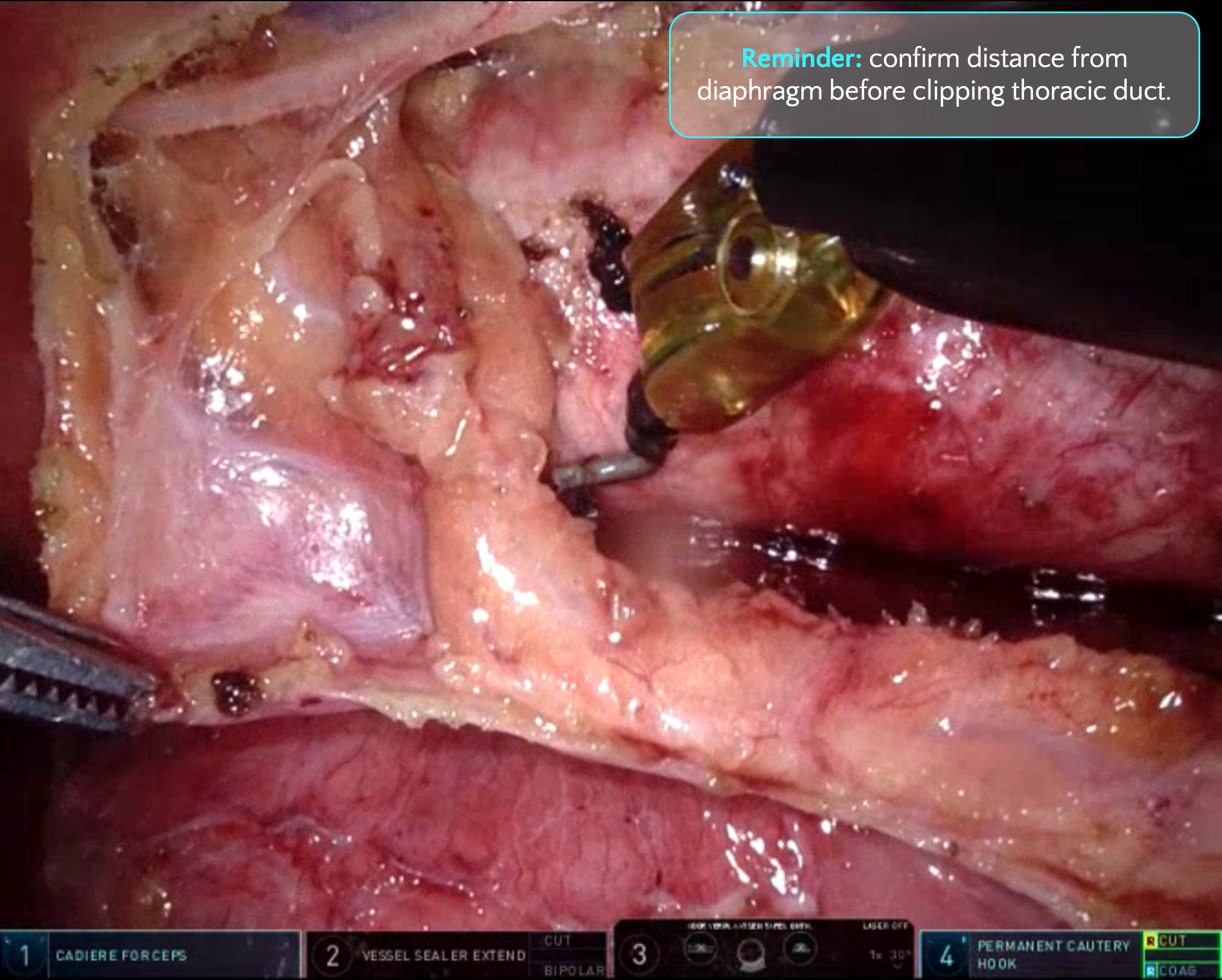
**

**Figure 11.** AI-assisted intraoperative image of thoracic duct dissection during RAMIE, emphasizing the need to verify safe clipping level relative to the diaphragm.
