# Supplementary material for: Are we developing the right intraoperative AI assistance? Surgeons’ perspectives and desired functions
Source: Surg Endosc. 2026 Apr 9;40(6):5259–66. doi: 10.1007/s00464-026-12791-9 (PMC13246846; doi:10.1007/s00464-026-12791-9)
Supplement: Supplementary file 6 — Supplementary file6 (DOCX 1338 kb) [file 464_2026_12791_MOESM6_ESM.docx]

**
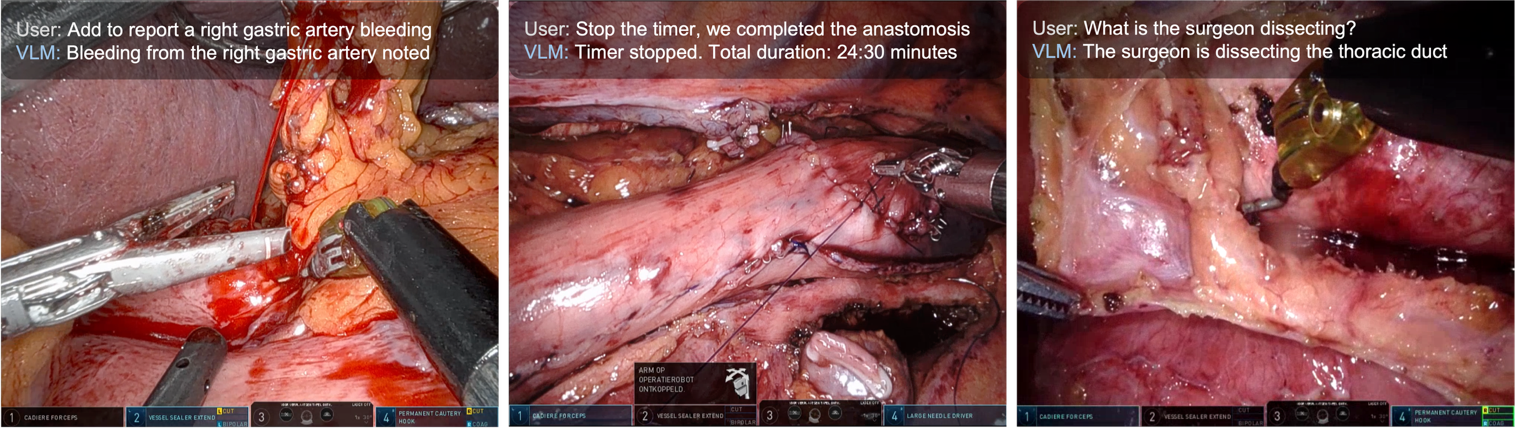
**

**Figure 12.** Examples of vision–language model assistance during RAMIE: operative report annotation, procedural step timing, and real-time response to user query.
